# A second polymorph of bis­(triphenyl-λ^5^-phosphanyl­idene)ammonium chloride–boric acid adduct

**DOI:** 10.1107/S1600536813020886

**Published:** 2013-08-17

**Authors:** Bruno A. Correia Bicho, Christoph Bolli, Carsten Jenne, Helene Seeger

**Affiliations:** a Fachbereich C - Anorganische Chemie, Bergische Universität Wuppertal, Gausssstrasse 20, 42119 Wuppertal, Germany

## Abstract

The title crystal structure is a new triclinic polymorph of [(Ph_3_P)_2_N]Cl·(B(OH)_3_) or C_36_H_30_NP_2_^+^·Cl^−^·BH_3_O_3_. The crystal structure of the ortho­rhom­bic polymorph was reported by [Andrews *et al.* (1983 ▶). *Acta Cryst.* C**39**, 880–882]. In the crystal, the [(Ph_3_P)_2_N]^+^ cations have no significant contacts to the chloride ions nor to the boric acid mol­ecules. This is indicated by the P—N—P angle of 137.28 (8)°, which is in the expected range for a free [(Ph_3_P)_2_N]^+^ cation. The boric acid mol­ecules form inversion dimers *via* pairs of O—H⋯O hydrogen bonds, and each boric acid mol­ecule forms two additional O—H⋯Cl hydrogen bonds to one chloride anion. These entities fill channels, created by the [(Ph_3_P)_2_N]^+^ cations, along the *c*-axis direction.

## Related literature

For the ortho­rhom­bic polymorph of the title compound, see: Andrews *et al.* (1983 ▶). Other bis­(tri­phenyl­phosphine)iminium halide structures include [(Ph_3_P)_2_N]Cl (Knapp & Uzun, 2010*a* ▶), [(Ph_3_P)_2_N]Br·CH_3_CN (Knapp & Uzun, 2010*b* ▶), [(Ph_3_P)_2_N]I (Beckett *et al.*, 2010 ▶) and [(Ph_3_P)_2_N][ClHCl] (Gellhaar & Knapp, 2011 ▶). For a discussion of the [(Ph_3_P)_2_N]^+^ cation, see: Lewis & Dance (2000 ▶). For a theoretical study on boric acid dimers, see: Larkin *et al.* (2006 ▶). For an overview of the different polymorphs of boric acid, see: Shuvalov & Burns (2003 ▶).
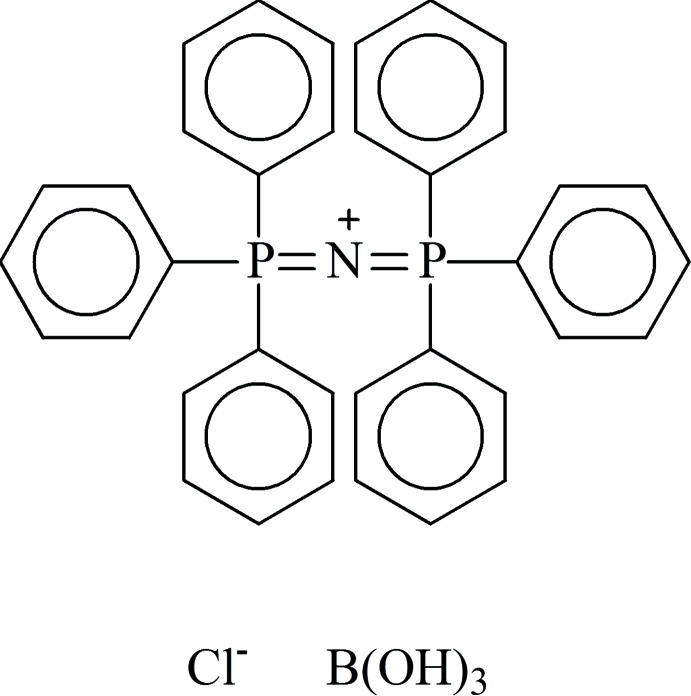


## Experimental

### 

#### Crystal data

C_36_H_30_NP_2_^+^·Cl^−^·BH_3_O_3_
*M* *_r_* = 635.83
Triclinic, 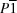

*a* = 10.7720 (2) Å
*b* = 11.4243 (3) Å
*c* = 14.3507 (4) Å
α = 107.244 (2)°
β = 105.648 (2)°
γ = 93.2742 (19)°
*V* = 1605.99 (7) Å^3^
*Z* = 2
Mo *K*α radiation
μ = 0.26 mm^−1^
*T* = 150 K
0.18 × 0.14 × 0.10 mm

#### Data collection

Agilent Xcalibur (Eos, Gemini ultra) diffractometer
Absorption correction: multi-scan (*CrysAlis PRO*; Agilent, 2013 ▶) *T*_min_ = 0.256, *T*_max_ = 1.000
14941 measured reflections
8731 independent reflections
6913 reflections with *I* > 2σ(*I*)
*R*_int_ = 0.023

#### Refinement

*R*[*F* ^2^ > 2σ(*F* ^2^)] = 0.041
*wR*(*F* ^2^) = 0.098
*S* = 1.04
8731 reflections
409 parameters
H atoms treated by a mixture of independent and constrained refinement
Δρ_max_ = 0.50 e Å^−3^
Δρ_min_ = −0.35 e Å^−3^

### 

Data collection: *CrysAlis PRO* (Agilent, 2013 ▶); cell refinement: *CrysAlis PRO*; data reduction: *CrysAlis PRO*; program(s) used to solve structure: *SHELXS97* (Sheldrick, 2008 ▶); program(s) used to refine structure: *SHELXL2013* (Sheldrick, 2008 ▶); molecular graphics: *DIAMOND* (Brandenburg *et al.*, 2012 ▶); software used to prepare material for publication: *OLEX2* (Dolomanov *et al.*, 2009 ▶).

## Supplementary Material

Crystal structure: contains datablock(s) I. DOI: 10.1107/S1600536813020886/su2629sup1.cif

Structure factors: contains datablock(s) I. DOI: 10.1107/S1600536813020886/su2629Isup2.hkl

Supplementary material file. DOI: 10.1107/S1600536813020886/su2629Isup3.cml

Additional supplementary materials:  crystallographic information; 3D view; checkCIF report

## Figures and Tables

**Table 1:** Hydrogen-bond geometry (Å, °)

*D*—H⋯*A*	*D*—H	H⋯*A*	*D*⋯*A*	*D*—H⋯*A*
O3—H3*O*⋯O2^i^	0.86 (3)	1.90 (3)	2.7585 (19)	180 (3)
O2—H2*O*⋯Cl1	0.79 (3)	2.30 (3)	3.0595 (14)	161 (3)
O1—H1*O*⋯Cl1	0.77 (3)	2.42 (3)	3.1757 (17)	166 (3)

Symmetry code: (i) 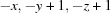.

**Fig. 1.**
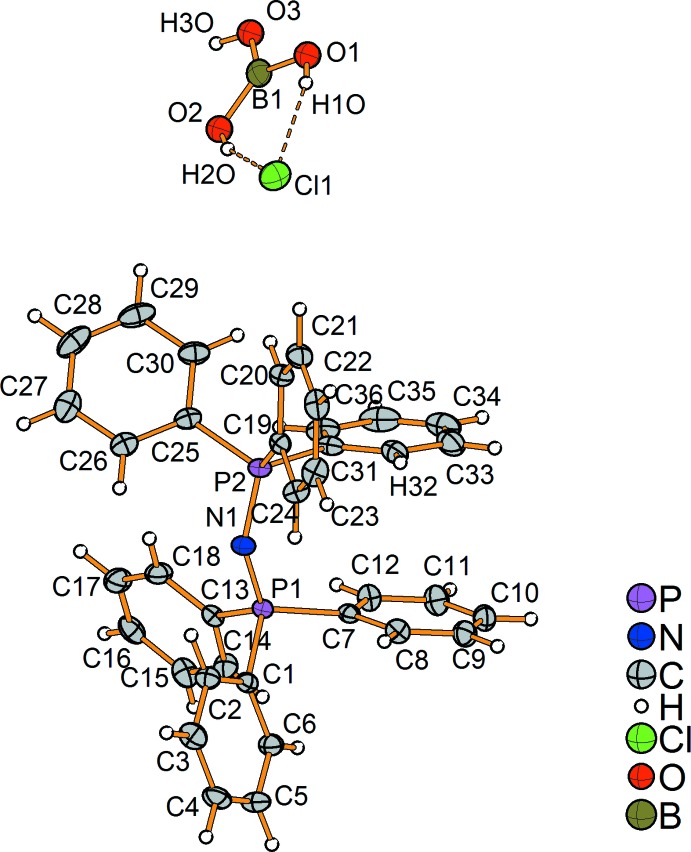
View of the molecular structure of the title compound, with atom labelling. Displacement ellipsoids are drawn at the 50% probability level. The O-H···Cl hydrogen bonds are shown as dashed lines (see Table 1 for details).

**Fig. 2.**
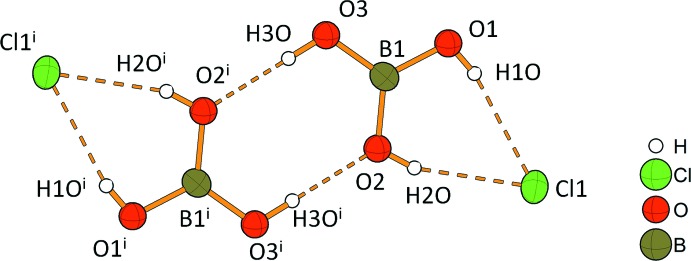
View of the dimeric moiety consisting of two chloride anions and two boric acid molecules, which is linked by O—H···O and O—H···Cl hydrogen bonds [dashed lines; see Table 1 for details; symmetry code: (i) -x, -y + 1, -z + 1].

## supplementary crystallographic information

### 1. Comment

Often the [(Ph_3_P)_2_N]^+^ cation is partnered by a bulky anion, while crystal structures containing small anions and especially halides are rare. Only very recently, the crystal structures of the solvate-free halides [(Ph_3_P)_2_N]I (Beckett *et al.*, 2010) and [(Ph_3_P)_2_N]Cl (Knapp & Uzun, 2010*a*) and the acetonitrile solvate [(Ph_3_P)_2_N]Br.CH_3_CN (Knapp & Uzun, 2010*b*) and the dichloride [(Ph_3_P)_2_N][ClHCl] (Gellhaar & Knapp, 2011) were published.

In the course of our investigations on the crystal structures of [(Ph_3_P)_2_N]^+^ salts with small counter anions, colourless single crystals of [(Ph_3_P)_2_N]Cl.(B(OH)_3_) were isolated from an acetonitrile/diethyl ether solution. This compound is formed by hydrolysis of [(Ph_3_P)_2_N][BCl_4_]. The dichloride [(Ph_3_P)_2_N][ClHCl] (Gellhaar & Knapp, 2011) was identified as a by-product of this hydrolysis reaction by single-crystal X-ray diffraction.

Surprisingly, the determined crystal structure of [(Ph_3_P)_2_N]Cl.(B(OH)_3_), Fig. 1, is distinctly different from the crystal structure (orthorhombic, *Pbca*, *a* = 19.010 (3), *b*= 18.869 (4), *c*= 18.432 (6) Å), which was reported previously for this compound (Andrews *et al.*, 1983) and thus represents a second polymorph. It can be assumed that different crystallization conditions, dichloromethane/n-hexane (Andrews *et al.*, 1983) *versus* acetonitrile/diethyl ether (this work), caused the crystallization of different polymorphs. The two polymorphs show different orientations of the phenyl groups of the [(Ph_3_P)_2_N]^+^ cation and a different orientation of the anionic and cationic parts relative to each other in the crystal lattice. The herein reported polymorph has a higher density (1.315 Mg m^-3^, *T* =150 K) than the structure reported by Andrews *et al.* (1.277 Mg m^-3^, *T*=291 k).

The bis(triphenyl-λ^5^-phosphanylidene)ammonium cation shows bond angles (P—N—P angle [137.28 (8)°]) and bond lengths (P—N (1.5836 (12) and 1.5839 (12) Å) and P—C distances (1.7951 (15)–1.8004 (16) Å) in the expected range (Lewis & Dance, 2000).

In the crystal, the boric acid molecules form inversion dimers *via* a pair of O—H···O hydrogen bonds (Fig. 2 and Table 1). Each boric acid molecule forms two additional O—H···Cl hydrogen bonds to one chloride ion (Fig. 2 and Table 1). This part of the crystal structure is very similar to the boric acid dimer in the orthorhombic polymorph.

### 2. Experimental

Single crystals of the title compound, suitable for X-ray diffraction, were obtained as an hydrolysis product of [(Ph_3_P)_2_N][BCl_4_] by layering an acetonitrile solution with diethyl ether.

### 3. Refinement

All hydrogen atoms attached to the aromatic rings were placed in calculated positions (C—H = 0.93 Å) and refined as riding atoms, with *U*_iso_(H) = 1.2 *U*_eq_(C). The oxygen-bonded H atoms were located in a difference Fourier map and freely refined.

### Crystal data

**Table:**

C_36_H_30_NP_2_^+^·Cl^−^·BH_3_O_3_	*Z* = 2
*M**_r_* = 635.83	*F*(000) = 664
Triclinic, *P*1	*D*_x_ = 1.315 Mg m^−^^3^
Hall symbol: -P 1	Mo *K*α radiation, λ = 0.71073 Å
*a* = 10.7720 (2) Å	Cell parameters from 6049 reflections
*b* = 11.4243 (3) Å	θ = 30.0–1.9°
*c* = 14.3507 (4) Å	µ = 0.26 mm^−^^1^
α = 107.244 (2)°	*T* = 150 K
β = 105.648 (2)°	Block, colourless
γ = 93.2742 (19)°	0.18 × 0.14 × 0.10 mm
*V* = 1605.99 (7) Å^3^	

### Data collection

**Table:**

Agilent Xcalibur (Eos, Gemini ultra) diffractometer	8731 independent reflections
Radiation source: fine-focus sealed tube	6913 reflections with *I* > 2σ(*I*)
Graphite monochromator	*R*_int_ = 0.023
Detector resolution: 16.2705 pixels mm^-1^	θ_max_ = 30.9°, θ_min_ = 1.9°
ω scans	*h* = −14→10
Absorption correction: multi-scan (*CrysAlis PRO*; Agilent, 2013)	*k* = −14→15
*T*_min_ = 0.256, *T*_max_ = 1.000	*l* = −20→20
14941 measured reflections	

### Refinement

**Table:**

Refinement on *F*^2^	Primary atom site location: structure-invariant direct methods
Least-squares matrix: full	Secondary atom site location: difference Fourier map
*R*[*F*^2^ > 2σ(*F*^2^)] = 0.041	Hydrogen site location: inferred from neighbouring sites
*wR*(*F*^2^) = 0.098	H atoms treated by a mixture of independent and constrained refinement
*S* = 1.04	*w* = 1/[σ^2^(*F*_o_^2^) + (0.0401*P*)^2^ + 0.4455*P*] where *P* = (*F*_o_^2^ + 2*F*_c_^2^)/3
8731 reflections	(Δ/σ)_max_ = 0.001
409 parameters	Δρ_max_ = 0.50 e Å^−^^3^
0 restraints	Δρ_min_ = −0.35 e Å^−^^3^

### Special details

**Table:**

Geometry. All e.s.d.'s (except the e.s.d. in the dihedral angle between two l.s. planes) are estimated using the full covariance matrix. The cell e.s.d.'s are taken into account individually in the estimation of e.s.d.'s in distances, angles and torsion angles; correlations between e.s.d.'s in cell parameters are only used when they are defined by crystal symmetry. An approximate (isotropic) treatment of cell e.s.d.'s is used for estimating e.s.d.'s involving l.s. planes.
Refinement. Refinement of *F*^2^ against ALL reflections. The weighted *R*-factor *wR* and goodness of fit *S* are based on *F*^2^, conventional *R*-factors *R* are based on *F*, with *F* set to zero for negative *F*^2^. The threshold expression of *F*^2^ > 2σ(*F*^2^) is used only for calculating *R*-factors(gt) *etc*. and is not relevant to the choice of reflections for refinement. *R*-factors based on *F*^2^ are statistically about twice as large as those based on *F*, and *R*- factors based on ALL data will be even larger. All hydrogen atoms attached to the aromatic rings were placed in calculated positions (C—H = 0.93 Å) and refined as riding atoms, with *U*_iso_(H) = 1.2 *U*_eq_(C). The oxygen-bonded hydrogen atoms were taken from the Fourier map and were refined isotropically.

### Fractional atomic coordinates and isotropic or equivalent isotropic displacement parameters (Å^2^)

**Table:**

	*x*	*y*	*z*	*U*_iso_*/*U*_eq_	
P2	0.64033 (4)	0.84597 (3)	0.28032 (3)	0.01673 (9)	
P1	0.49238 (4)	0.60975 (3)	0.26011 (3)	0.01533 (8)	
N1	0.61330 (12)	0.70542 (11)	0.27330 (10)	0.0187 (3)	
C7	0.34257 (14)	0.61496 (13)	0.16954 (11)	0.0171 (3)	
C19	0.73612 (14)	0.85848 (13)	0.19766 (11)	0.0177 (3)	
C25	0.73552 (15)	0.94085 (14)	0.40702 (11)	0.0206 (3)	
C1	0.53257 (14)	0.45674 (13)	0.21385 (11)	0.0173 (3)	
C13	0.45827 (14)	0.62377 (13)	0.37872 (11)	0.0178 (3)	
C6	0.43562 (16)	0.35398 (14)	0.17729 (12)	0.0229 (3)	
H6	0.3478	0.3649	0.1734	0.027*	
C21	0.86818 (16)	0.98473 (16)	0.14146 (13)	0.0263 (3)	
H21	0.9109	1.0634	0.1486	0.032*	
C10	0.11519 (16)	0.62679 (15)	0.02598 (12)	0.0253 (3)	
H10	0.0378	0.6313	−0.0230	0.030*	
C8	0.32793 (15)	0.56684 (14)	0.06508 (12)	0.0217 (3)	
H8	0.3962	0.5303	0.0427	0.026*	
C24	0.74587 (15)	0.75343 (14)	0.12204 (12)	0.0225 (3)	
H24	0.7052	0.6743	0.1157	0.027*	
C20	0.79778 (15)	0.97397 (14)	0.20726 (12)	0.0219 (3)	
H20	0.7916	1.0457	0.2592	0.026*	
C26	0.82218 (15)	0.88565 (15)	0.46632 (12)	0.0237 (3)	
H26	0.8255	0.7994	0.4412	0.028*	
C12	0.24218 (15)	0.66948 (15)	0.20093 (12)	0.0225 (3)	
H12	0.2513	0.7033	0.2718	0.027*	
C3	0.69251 (16)	0.32136 (15)	0.18846 (13)	0.0265 (3)	
H3	0.7801	0.3098	0.1918	0.032*	
C14	0.35051 (15)	0.55110 (15)	0.38051 (12)	0.0242 (3)	
H14	0.2924	0.4959	0.3186	0.029*	
C4	0.59580 (17)	0.21979 (14)	0.15264 (13)	0.0275 (4)	
H4	0.6174	0.1385	0.1320	0.033*	
C32	0.43908 (15)	0.89028 (15)	0.13559 (13)	0.0252 (3)	
H32	0.4843	0.8532	0.0887	0.030*	
C2	0.66115 (15)	0.44051 (14)	0.21962 (12)	0.0212 (3)	
H2	0.7273	0.5105	0.2447	0.025*	
C18	0.54362 (17)	0.70184 (14)	0.46993 (12)	0.0261 (3)	
H18	0.6175	0.7508	0.4693	0.031*	
C16	0.41352 (17)	0.63913 (15)	0.56316 (13)	0.0271 (3)	
H16	0.3973	0.6457	0.6262	0.033*	
C11	0.12891 (16)	0.67475 (16)	0.12927 (13)	0.0274 (3)	
H11	0.0604	0.7115	0.1512	0.033*	
C15	0.32914 (16)	0.56022 (16)	0.47325 (13)	0.0275 (3)	
H15	0.2556	0.5115	0.4747	0.033*	
C23	0.81557 (15)	0.76481 (16)	0.05559 (12)	0.0261 (3)	
H23	0.8217	0.6933	0.0034	0.031*	
C31	0.49389 (15)	0.90944 (13)	0.23950 (12)	0.0211 (3)	
C36	0.42608 (17)	0.96328 (15)	0.30771 (14)	0.0295 (4)	
H36	0.4609	0.9743	0.3785	0.035*	
C9	0.21448 (16)	0.57221 (15)	−0.00586 (12)	0.0255 (3)	
H9	0.2046	0.5382	−0.0769	0.031*	
C22	0.87572 (15)	0.87948 (16)	0.06513 (13)	0.0266 (3)	
H22	0.9226	0.8865	0.0192	0.032*	
C29	0.81699 (19)	1.13927 (16)	0.53898 (14)	0.0339 (4)	
H29	0.8165	1.2261	0.5637	0.041*	
C28	0.90074 (18)	1.08346 (18)	0.59779 (14)	0.0364 (4)	
H28	0.9569	1.1320	0.6633	0.044*	
C5	0.46804 (17)	0.23583 (14)	0.14666 (13)	0.0277 (4)	
H5	0.4022	0.1656	0.1214	0.033*	
C27	0.90334 (17)	0.95721 (18)	0.56189 (13)	0.0320 (4)	
H27	0.9610	0.9195	0.6030	0.038*	
C30	0.73351 (17)	1.06866 (14)	0.44378 (13)	0.0267 (3)	
H30	0.6752	1.1069	0.4036	0.032*	
C17	0.52151 (19)	0.70874 (15)	0.56217 (13)	0.0310 (4)	
H17	0.5809	0.7615	0.6246	0.037*	
C35	0.30674 (18)	1.00078 (16)	0.27085 (18)	0.0386 (5)	
H35	0.2615	1.0397	0.3173	0.046*	
C34	0.25385 (17)	0.98210 (16)	0.16809 (17)	0.0383 (5)	
H34	0.1727	1.0084	0.1439	0.046*	
C33	0.31852 (17)	0.92524 (17)	0.10030 (16)	0.0337 (4)	
H33	0.2805	0.9099	0.0291	0.040*	
Cl1	0.08761 (5)	0.28422 (4)	0.19113 (3)	0.03497 (11)	
O2	0.01672 (13)	0.41861 (12)	0.38407 (10)	0.0308 (3)	
O3	−0.04705 (13)	0.61727 (12)	0.42973 (11)	0.0362 (3)	
O1	−0.00992 (14)	0.54155 (15)	0.27226 (11)	0.0380 (3)	
B1	−0.01301 (18)	0.52440 (19)	0.36165 (15)	0.0277 (4)	
H2O	0.036 (2)	0.370 (2)	0.340 (2)	0.063 (8)*	
H1O	0.009 (3)	0.481 (3)	0.243 (2)	0.065 (9)*	
H3O	−0.038 (2)	0.606 (2)	0.487 (2)	0.066 (8)*	

### Atomic displacement parameters (Å^2^)

**Table:**

	*U*^11^	*U*^22^	*U*^33^	*U*^12^	*U*^13^	*U*^23^
P2	0.01776 (18)	0.01352 (17)	0.02030 (19)	0.00226 (13)	0.00854 (15)	0.00497 (14)
P1	0.01602 (18)	0.01386 (16)	0.01754 (18)	0.00255 (13)	0.00670 (14)	0.00562 (13)
N1	0.0181 (6)	0.0151 (6)	0.0242 (6)	0.0017 (5)	0.0081 (5)	0.0069 (5)
C7	0.0170 (7)	0.0149 (6)	0.0208 (7)	0.0015 (5)	0.0064 (6)	0.0076 (5)
C19	0.0159 (7)	0.0189 (7)	0.0203 (7)	0.0039 (5)	0.0065 (6)	0.0080 (6)
C25	0.0227 (8)	0.0183 (7)	0.0215 (7)	−0.0009 (6)	0.0119 (6)	0.0033 (6)
C1	0.0201 (7)	0.0158 (6)	0.0176 (7)	0.0029 (5)	0.0069 (6)	0.0065 (5)
C13	0.0197 (7)	0.0173 (7)	0.0198 (7)	0.0060 (5)	0.0081 (6)	0.0086 (6)
C6	0.0221 (8)	0.0191 (7)	0.0272 (8)	0.0013 (6)	0.0073 (6)	0.0075 (6)
C21	0.0246 (8)	0.0280 (8)	0.0301 (9)	0.0000 (6)	0.0090 (7)	0.0149 (7)
C10	0.0215 (8)	0.0283 (8)	0.0256 (8)	0.0030 (6)	0.0031 (7)	0.0118 (7)
C8	0.0243 (8)	0.0222 (7)	0.0212 (7)	0.0064 (6)	0.0105 (6)	0.0071 (6)
C24	0.0208 (7)	0.0211 (7)	0.0250 (8)	0.0024 (6)	0.0095 (6)	0.0044 (6)
C20	0.0231 (8)	0.0198 (7)	0.0243 (8)	0.0021 (6)	0.0081 (6)	0.0086 (6)
C26	0.0226 (8)	0.0237 (8)	0.0248 (8)	0.0003 (6)	0.0108 (6)	0.0051 (6)
C12	0.0221 (8)	0.0272 (8)	0.0206 (7)	0.0071 (6)	0.0089 (6)	0.0084 (6)
C3	0.0264 (8)	0.0263 (8)	0.0317 (9)	0.0113 (7)	0.0141 (7)	0.0104 (7)
C14	0.0195 (7)	0.0304 (8)	0.0240 (8)	0.0008 (6)	0.0067 (6)	0.0109 (7)
C4	0.0391 (10)	0.0159 (7)	0.0297 (9)	0.0104 (7)	0.0134 (8)	0.0066 (6)
C32	0.0217 (8)	0.0224 (7)	0.0354 (9)	0.0054 (6)	0.0098 (7)	0.0138 (7)
C2	0.0205 (7)	0.0198 (7)	0.0247 (8)	0.0032 (6)	0.0088 (6)	0.0073 (6)
C18	0.0329 (9)	0.0198 (7)	0.0246 (8)	−0.0035 (6)	0.0090 (7)	0.0067 (6)
C16	0.0387 (10)	0.0288 (8)	0.0242 (8)	0.0148 (7)	0.0174 (7)	0.0146 (7)
C11	0.0217 (8)	0.0353 (9)	0.0290 (9)	0.0107 (7)	0.0097 (7)	0.0126 (7)
C15	0.0235 (8)	0.0371 (9)	0.0307 (9)	0.0070 (7)	0.0129 (7)	0.0190 (7)
C23	0.0217 (8)	0.0320 (8)	0.0251 (8)	0.0071 (7)	0.0111 (7)	0.0058 (7)
C31	0.0203 (7)	0.0145 (6)	0.0319 (8)	0.0035 (5)	0.0122 (7)	0.0080 (6)
C36	0.0273 (9)	0.0231 (8)	0.0404 (10)	0.0021 (7)	0.0186 (8)	0.0064 (7)
C9	0.0313 (9)	0.0259 (8)	0.0196 (8)	0.0051 (7)	0.0074 (7)	0.0077 (6)
C22	0.0201 (8)	0.0396 (9)	0.0264 (8)	0.0059 (7)	0.0109 (7)	0.0160 (7)
C29	0.0424 (11)	0.0232 (8)	0.0322 (9)	−0.0050 (7)	0.0223 (8)	−0.0050 (7)
C28	0.0334 (10)	0.0421 (10)	0.0231 (9)	−0.0109 (8)	0.0128 (8)	−0.0055 (8)
C5	0.0313 (9)	0.0164 (7)	0.0326 (9)	−0.0011 (6)	0.0086 (7)	0.0058 (6)
C27	0.0265 (9)	0.0433 (10)	0.0245 (8)	−0.0006 (7)	0.0084 (7)	0.0088 (7)
C30	0.0313 (9)	0.0202 (7)	0.0301 (9)	0.0017 (6)	0.0165 (7)	0.0042 (6)
C17	0.0456 (11)	0.0235 (8)	0.0199 (8)	−0.0004 (7)	0.0083 (8)	0.0040 (6)
C35	0.0281 (9)	0.0234 (8)	0.0701 (14)	0.0062 (7)	0.0302 (10)	0.0091 (9)
C34	0.0204 (8)	0.0271 (9)	0.0725 (15)	0.0071 (7)	0.0147 (9)	0.0223 (9)
C33	0.0240 (8)	0.0301 (9)	0.0498 (11)	0.0045 (7)	0.0061 (8)	0.0213 (8)
Cl1	0.0384 (2)	0.0343 (2)	0.0308 (2)	0.00153 (18)	0.01415 (19)	0.00595 (18)
O2	0.0378 (7)	0.0278 (6)	0.0289 (7)	0.0064 (5)	0.0132 (6)	0.0089 (5)
O3	0.0450 (8)	0.0345 (7)	0.0383 (8)	0.0161 (6)	0.0178 (6)	0.0185 (6)
O1	0.0394 (8)	0.0480 (9)	0.0370 (8)	0.0125 (7)	0.0168 (6)	0.0230 (7)
B1	0.0202 (9)	0.0330 (10)	0.0306 (10)	0.0019 (7)	0.0061 (8)	0.0132 (8)

### Geometric parameters (Å, º)

**Table:**

P1—N1	1.5839 (12)	C4—C5	1.382 (2)
P2—N1	1.5836 (12)	C32—H32	0.9500
P2—C19	1.7990 (15)	C32—C31	1.392 (2)
P2—C25	1.7976 (16)	C32—C33	1.388 (2)
P2—C31	1.8004 (16)	C2—H2	0.9500
P1—C7	1.7951 (15)	C18—H18	0.9500
P1—C1	1.7993 (14)	C18—C17	1.388 (2)
P1—C13	1.8001 (14)	C16—H16	0.9500
C7—C8	1.396 (2)	C16—C15	1.374 (2)
C7—C12	1.391 (2)	C16—C17	1.377 (2)
C19—C24	1.390 (2)	C11—H11	0.9500
C19—C20	1.394 (2)	C15—H15	0.9500
C25—C26	1.396 (2)	C23—H23	0.9500
C25—C30	1.401 (2)	C23—C22	1.380 (2)
C1—C6	1.394 (2)	C31—C36	1.396 (2)
C1—C2	1.391 (2)	C36—H36	0.9500
C13—C14	1.399 (2)	C36—C35	1.395 (3)
C13—C18	1.385 (2)	C9—H9	0.9500
C6—H6	0.9500	C22—H22	0.9500
C6—C5	1.386 (2)	C29—H29	0.9500
C21—H21	0.9500	C29—C28	1.381 (3)
C21—C20	1.388 (2)	C29—C30	1.388 (2)
C21—C22	1.389 (2)	C28—H28	0.9500
C10—H10	0.9500	C28—C27	1.385 (3)
C10—C11	1.382 (2)	C5—H5	0.9500
C10—C9	1.383 (2)	C27—H27	0.9500
C8—H8	0.9500	C30—H30	0.9500
C8—C9	1.381 (2)	C17—H17	0.9500
C24—H24	0.9500	C35—H35	0.9500
C24—C23	1.392 (2)	C35—C34	1.375 (3)
C20—H20	0.9500	C34—H34	0.9500
C26—H26	0.9500	C34—C33	1.377 (3)
C26—C27	1.386 (2)	C33—H33	0.9500
C12—H12	0.9500	Cl1—H2O	2.30 (3)
C12—C11	1.385 (2)	Cl1—H1O	2.42 (3)
C3—H3	0.9500	O1—B1	1.362 (2)
C3—C4	1.384 (2)	O2—B1	1.373 (2)
C3—C2	1.393 (2)	O2—H2O	0.79 (3)
C14—H14	0.9500	O3—B1	1.356 (2)
C14—C15	1.386 (2)	O3—H3O	0.86 (3)
C4—H4	0.9500	O1—H1O	0.77 (3)
			
N1—P2—C19	109.24 (7)	C1—C2—C3	119.79 (14)
N1—P2—C25	111.46 (7)	C1—C2—H2	120.1
N1—P2—C31	113.23 (7)	C3—C2—H2	120.1
C19—P2—C31	106.52 (7)	C13—C18—H18	119.9
C25—P2—C19	105.98 (7)	C13—C18—C17	120.21 (15)
C25—P2—C31	110.02 (7)	C17—C18—H18	119.9
N1—P1—C7	115.63 (7)	C15—C16—H16	119.9
N1—P1—C1	107.25 (7)	C15—C16—C17	120.25 (15)
N1—P1—C13	112.91 (7)	C17—C16—H16	119.9
C7—P1—C1	106.21 (7)	C10—C11—C12	120.26 (15)
C7—P1—C13	107.10 (7)	C10—C11—H11	119.9
C1—P1—C13	107.25 (6)	C12—C11—H11	119.9
P1—N1—P2	137.28 (8)	C14—C15—H15	119.7
C8—C7—P1	119.31 (11)	C16—C15—C14	120.51 (15)
C12—C7—P1	121.57 (11)	C16—C15—H15	119.7
C12—C7—C8	119.08 (14)	C24—C23—H23	119.9
C24—C19—P2	120.08 (11)	C22—C23—C24	120.30 (15)
C24—C19—C20	119.75 (13)	C22—C23—H23	119.9
C20—C19—P2	120.15 (11)	C32—C31—P2	118.76 (12)
C26—C25—P2	117.86 (11)	C32—C31—C36	119.40 (15)
C26—C25—C30	119.66 (15)	C36—C31—P2	121.35 (13)
C30—C25—P2	122.24 (13)	C31—C36—H36	120.3
C6—C1—P1	119.74 (11)	C35—C36—C31	119.35 (18)
C2—C1—P1	120.12 (11)	C35—C36—H36	120.3
C2—C1—C6	120.05 (14)	C10—C9—H9	119.8
C14—C13—P1	120.66 (12)	C8—C9—C10	120.32 (15)
C18—C13—P1	119.79 (11)	C8—C9—H9	119.8
C18—C13—C14	119.44 (14)	C21—C22—H22	119.8
C1—C6—H6	120.2	C23—C22—C21	120.45 (14)
C5—C6—C1	119.65 (15)	C23—C22—H22	119.8
C5—C6—H6	120.2	C28—C29—H29	120.0
C20—C21—H21	120.3	C28—C29—C30	120.07 (16)
C20—C21—C22	119.44 (15)	C30—C29—H29	120.0
C22—C21—H21	120.3	C29—C28—H28	119.8
C11—C10—H10	120.1	C29—C28—C27	120.40 (17)
C11—C10—C9	119.81 (15)	C27—C28—H28	119.8
C9—C10—H10	120.1	C6—C5—H5	119.9
C7—C8—H8	119.9	C4—C5—C6	120.27 (15)
C9—C8—C7	120.24 (14)	C4—C5—H5	119.9
C9—C8—H8	119.9	C26—C27—H27	119.9
C19—C24—H24	120.2	C28—C27—C26	120.29 (18)
C19—C24—C23	119.66 (14)	C28—C27—H27	119.9
C23—C24—H24	120.2	C25—C30—H30	120.1
C19—C20—H20	119.8	C29—C30—C25	119.83 (17)
C21—C20—C19	120.40 (14)	C29—C30—H30	120.1
C21—C20—H20	119.8	C18—C17—H17	120.0
C25—C26—H26	120.1	C16—C17—C18	120.01 (16)
C27—C26—C25	119.73 (16)	C16—C17—H17	120.0
C27—C26—H26	120.1	C36—C35—H35	119.6
C7—C12—H12	119.9	C34—C35—C36	120.74 (17)
C11—C12—C7	120.28 (14)	C34—C35—H35	119.6
C11—C12—H12	119.9	C35—C34—H34	120.0
C4—C3—H3	120.1	C35—C34—C33	119.96 (17)
C4—C3—C2	119.82 (15)	C33—C34—H34	120.0
C2—C3—H3	120.1	C32—C33—H33	119.9
C13—C14—H14	120.2	C34—C33—C32	120.27 (18)
C15—C14—C13	119.55 (15)	C34—C33—H33	119.9
C15—C14—H14	120.2	H2O—Cl1—H1O	53.1 (9)
C3—C4—H4	119.8	B1—O2—H2O	112.3 (19)
C5—C4—C3	120.42 (14)	B1—O3—H3O	113.2 (17)
C5—C4—H4	119.8	B1—O1—H1O	103 (2)
C31—C32—H32	119.9	O3—B1—O2	120.11 (16)
C33—C32—H32	119.9	O3—B1—O1	117.20 (17)
C33—C32—C31	120.22 (16)	O1—B1—O2	122.69 (17)
			
P2—C19—C24—C23	177.23 (12)	C1—P1—C13—C14	−65.54 (14)
P2—C19—C20—C21	−177.83 (12)	C1—P1—C13—C18	110.61 (13)
P2—C25—C26—C27	175.93 (12)	C1—C6—C5—C4	0.3 (2)
P2—C25—C30—C29	−174.63 (12)	C13—P1—N1—P2	−82.61 (13)
P2—C31—C36—C35	−174.05 (12)	C13—P1—C7—C8	−159.22 (11)
P1—C7—C8—C9	−178.67 (12)	C13—P1—C7—C12	22.92 (14)
P1—C7—C12—C11	178.46 (12)	C13—P1—C1—C6	70.30 (13)
P1—C1—C6—C5	−176.66 (12)	C13—P1—C1—C2	−106.26 (13)
P1—C1—C2—C3	176.75 (12)	C13—C14—C15—C16	−0.6 (2)
P1—C13—C14—C15	177.56 (12)	C13—C18—C17—C16	−0.9 (3)
P1—C13—C18—C17	−176.87 (13)	C6—C1—C2—C3	0.2 (2)
N1—P2—C19—C24	16.53 (15)	C8—C7—C12—C11	0.6 (2)
N1—P2—C19—C20	−165.31 (12)	C24—C19—C20—C21	0.3 (2)
N1—P2—C25—C26	29.30 (14)	C24—C23—C22—C21	0.4 (3)
N1—P2—C25—C30	−156.36 (12)	C20—C19—C24—C23	−0.9 (2)
N1—P2—C31—C32	−84.03 (13)	C20—C21—C22—C23	−1.0 (2)
N1—P2—C31—C36	87.85 (14)	C26—C25—C30—C29	−0.4 (2)
N1—P1—C7—C8	73.96 (13)	C12—C7—C8—C9	−0.8 (2)
N1—P1—C7—C12	−103.90 (13)	C3—C4—C5—C6	−0.5 (3)
N1—P1—C1—C6	−168.15 (12)	C14—C13—C18—C17	−0.7 (2)
N1—P1—C1—C2	15.29 (14)	C4—C3—C2—C1	−0.5 (2)
N1—P1—C13—C14	176.53 (12)	C32—C31—C36—C35	−2.2 (2)
N1—P1—C13—C18	−7.31 (15)	C2—C1—C6—C5	−0.1 (2)
C7—P1—N1—P2	41.23 (15)	C2—C3—C4—C5	0.6 (2)
C7—P1—C1—C6	−43.96 (13)	C18—C13—C14—C15	1.4 (2)
C7—P1—C1—C2	139.48 (12)	C11—C10—C9—C8	−0.8 (2)
C7—P1—C13—C14	48.12 (14)	C15—C16—C17—C18	1.7 (3)
C7—P1—C13—C18	−135.73 (13)	C31—P2—N1—P1	−14.74 (15)
C7—C8—C9—C10	0.9 (2)	C31—P2—C19—C24	−106.11 (13)
C7—C12—C11—C10	−0.6 (2)	C31—P2—C19—C20	72.05 (14)
C19—P2—N1—P1	−133.27 (12)	C31—P2—C25—C26	155.77 (12)
C19—P2—C25—C26	−89.45 (13)	C31—P2—C25—C30	−29.89 (15)
C19—P2—C25—C30	84.89 (14)	C31—C32—C33—C34	1.7 (2)
C19—P2—C31—C32	36.06 (14)	C31—C36—C35—C34	1.8 (3)
C19—P2—C31—C36	−152.05 (12)	C36—C35—C34—C33	0.3 (3)
C19—C24—C23—C22	0.6 (2)	C9—C10—C11—C12	0.7 (3)
C25—P2—N1—P1	109.95 (13)	C22—C21—C20—C19	0.6 (2)
C25—P2—C19—C24	136.73 (13)	C29—C28—C27—C26	0.2 (3)
C25—P2—C19—C20	−45.10 (14)	C28—C29—C30—C25	−0.7 (2)
C25—P2—C31—C32	150.50 (12)	C30—C25—C26—C27	1.4 (2)
C25—P2—C31—C36	−37.61 (14)	C30—C29—C28—C27	0.8 (3)
C25—C26—C27—C28	−1.4 (2)	C17—C16—C15—C14	−1.0 (3)
C1—P1—N1—P2	159.47 (11)	C35—C34—C33—C32	−2.1 (3)
C1—P1—C7—C8	−44.85 (13)	C33—C32—C31—P2	172.50 (12)
C1—P1—C7—C12	137.28 (12)	C33—C32—C31—C36	0.5 (2)

### Hydrogen-bond geometry (Å, º)

**Table:**

*D*—H···*A*	*D*—H	H···*A*	*D*···*A*	*D*—H···*A*
O3—H3*O*···O2^i^	0.86 (3)	1.90 (3)	2.7585 (19)	180 (3)
O2—H2*O*···Cl1	0.79 (3)	2.30 (3)	3.0595 (14)	161 (3)
O1—H1*O*···Cl1	0.77 (3)	2.42 (3)	3.1757 (17)	166 (3)

Symmetry code: (i) −*x*, −*y*+1, −*z*+1.
